# Presence and significance of *Helicobacter spp.* in the gastric mucosa of Portuguese dogs

**DOI:** 10.1186/s13099-015-0057-1

**Published:** 2015-04-16

**Authors:** Irina Amorim, Annemieke Smet, Odete Alves, Silvia Teixeira, Ana Laura Saraiva, Marian Taulescu, Celso Reis, Freddy Haesebrouck, Fátima Gärtner

**Affiliations:** Instituto de Investigação e Inovação em Saúde, Universidade do Porto, Porto, Portugal; IPATIMUP - Institute of Molecular Pathology and Immunology of the University of Porto, Rua Dr. Roberto Frias s/n, 4200-465 Porto, Portugal; Institute of Biomedical Sciences Abel Salazar (ICBAS), University of Porto, Rua Jorge Viterbo Ferreira nr.228, 4050-313 Porto, Portugal; Faculty of Veterinary Medicine, Ghent University, Salisburylaan 133, Merelbeke, B-9820 Belgium; CECAV, Centro de Ciência Animal e Veterinária, Universidade de Trás-os-Montes e Alto Douro, Quinta de Prados, 5000-801 Vila Real, Portugal; Faculty of Veterinary Medicine, University of Agricultural Sciences and Veterinary Medicine, Cluj-Napoca, Romania; Faculty of Medicine, University of Porto, Alameda Prof. Hernâni Monteiro, 4200-319 Porto, Portugal

**Keywords:** Canine gastric mucosa, Dogs, Non-*Helicobacter pylori* helicobacters (NHPH), Histochemistry, Immunohistochemistry (IHC), Polymerase chain reaction (PCR), Stomach

## Abstract

**Background:**

Non-*Helicobacter pylori* Helicobacters (NHPH) are also able to cause disease in humans. Dogs are a natural reservoir for many of these species. Close and intense human contact with animals has been identified as a risk factor and therefore, an important zoonotic significance has been attributed to NHPH.

**Methods:**

To determine the prevalence of *Helicobacter* species and the gastric histopathological changes associated, gastric mucosa samples of 69 dogs were evaluated.

**Results:**

Only one dog presented a normal histopathological mucosa with absence of spiral-shaped organisms. A normal gastric mucosa and the presence of spiral-shaped bacteria was observed in two dogs. All remaining animals presented histopathological changes representative of gastritis. *Helicobacter* species were detected in 60 dogs (87.0%) by at least one detection method. Histological, histochemical and immunohistochemical evaluations revealed that *Helicobacter* spp. were present in 45 (65.2%), 52 (75.4%) and 57 (82.6%) dogs, respectively. Spiral-shaped bacteria were detected by qPCR analysis in 33 (47.8%) dogs. *H. heilmannii-*like organisms were identified in 22 animals (66.7%) and predominantly in the antral gastric region. *H. salomonis* was the second most prevalent species (51.5%) although it was mainly found in association with other *Helicobacter* spp. and in the body gastric region. *H. bizzozeronii and H. felis* were less frequently detected.

**Conclusions:**

It was concluded that, despite the high incidence and worldwide distribution of gastric NHPH in dogs, the presence of specific *Helicobacter* species may vary between geographic regions. NHPH infections were significantly accompanied by mild to moderate intraepithelial lymphocyte infiltration and mild to moderate gastric epithelial injury, but a clear relationship between gastritis and *Helicobacter* infection could not be established.

## Introduction

The genus *Helicobacter* is composed of at least 40 species [1]. Among these, *H. pylori* is considered as an important pathogen whose natural host is man but its presence in the canine stomach has been rarely reported [2-4].

A large number of non-*Helicobacter pylori Helicobacter* species (NHPH) have also been recognized in humans and in several animals. Previously, NHPH were generally referred to as *H. heilmannii sensu lato* (s.l.) and included *H. suis*, a species colonizing the stomachs of pigs and a group of species known to colonize the gastric mucosa of dogs and cats: *H. felis*, *H. bizzozeronii*, *H. salomonis*, *H. cynogastricus*, *H. baculiformis* and *H. heilmannii sensu stricto* (s.s.) [5]. Most of these gastric NHPH are also able to cause disease in humans [6,7]. Close and intense human contact with animals has been identified as a risk factor and therefore, an important zoonotic significance has been attributed to NHPH [6,8,9].

In pet animals, gastric *Helicobacter* spp. have been frequently described with a prevalence ranging from 67–86% in clinically healthy dogs and 61–100% in animals presenting chronic vomiting [10-14]. These microorganisms were detected in the stomach of about 100% of laboratory Beagle dogs and dogs from local shelters [15-17]. The predominant gastric *Helicobacter spp.* in cats and dogs are *H. felis*, *H. bizzozeronii* and *H. heilmannii sensu stricto* (*s.s.*), while *H. salomonis* is less often detected and the prevalence of *H. cynogastricus* and *H. baculiformis* has not yet been studied [9,18-20]. Mixed infections with different species can also occur [3,19].

While many studies have reported that the fundus and body have higher bacterial density and a higher probability of finding *Helicobacter* spp. [17,21,22] others have found no significant differences between the density of NHPH in the fundus, body and antrum [2,23-26] of the canine stomach. The discrepancies in these results can be attributed to the different laboratory diagnostic methodologies used by the various research groups.

The diagnostic methods used for *Helicobacter* spp*.* can be non-invasive and invasive [24]. Non-invasive methods like serology or detection of bacterial DNA and antigens in stools do not require a gastric biopsy or anaesthesia. The invasive methods, like bacterial cultures, histopathology, smears, electron microscopy or polymerase chain reaction (PCR) may require a gastric biopsy, which is frequently obtained through endoscopy under anaesthesia or by necropsy. Typically *Helicobacter* organisms are not easily visualized with the haematoxylin and eosin (HE) stain and so, their direct observation in biopsied specimens is highlighted by the use of special stains, such as the modified Giemsa (MG) stain. More elaborate and sensitive *Helicobacter*-detection methods such as immunohistochemistry (IHC) or polymerase chain reaction (PCR) are research tools rarely used in a diagnostic setting.

Several investigations have discussed the prevalence of *Helicobacter spp.* in dogs [2,10,11,14,16] but in only few studies the specific species present in the canine stomach were determined [4,19,27,28]. The accurate identification of the gastric helicobacters to the species level is essential in order to determine the prevalence and clinical significance of all taxa. The aim of this study was to determine the prevalence of different gastric *Helicobacter* species present in distinct stomach regions of the canine stomach (body and antrum) using histological, histochemical, immunohistochemical and molecular diagnostic techniques. The degree of colonization was characterized and correlated with the respective histopathological changes in the canine gastric mucosa.

## Methods

### Sample collection

Gastric tissues were obtained from 69 dogs (45 male and 24 female, ranging in age from 3 months to 15 years). The samples were randomly selected from the archives of the Laboratory of Veterinary Pathology, ICBAS-UP (Portugal) where they were received between 2010 and 2013. Samples were obtained from 20 dogs during endoscopic procedures, from five during surgery and from 44 dogs during necropsy examinations. All the procedures (surgical excision and necropsy examination) were performed in a clinical context attempting to treat the animals based on the best clinical judgment of their attending practitioners. The use of the excised tissues for research was explained to the owners and an informed consent was obtained for each case. None of the actions were taken solely for research purposes and the investigators had no influence on the selection and execution of such procedures. Only gastric samples in good condition of preservation were included in this study.

Tissues were fixed in 10% neutral buffered formalin and embedded in paraffin wax. Three consecutive sections of 3 μm were made, one being stained with HE, another with a MG stain and the third was used for immunohistochemical staining.

### Sample evaluation

The gastric samples were evaluated independently by two observers (IA and FG). Histopathological parameters such as alterations in cellularity, fibrosis of the *lamina propria* and gland atrophy were analysed according to the World Small Animal Veterinary Association (WSAVA) guidelines [29]. The degree of morphological features and inflammatory changes was graded as normal, mild, moderate or marked by using the available WSAVA gastrointestinal standardization visual analogue [29].

The microscopic evaluation was performed by analysing the entire section of the gastric tissue. The presence of *Helicobacter* spp*.* was assessed by HE and MG stains and by IHC. A dog was classified as *Helicobacter* positive when one of these methods gave a positive result. Additionally, bacterial density colonization was quantified: +, few organisms (<10 organisms/400x); ++, moderate number of organisms (10 to 50 organisms/400x); +++, large number of organisms (>50 organisms/400x) [24].

### Immunohistochemistry

For the immunohistochemical study, sections were deparaffinised, hydrated and antigen retrieval was performed in a pressure cooker in 10 mmol/L sodium citrate buffer, pH 6.0, for 2 minutes (min). Slides were cooled for 10 min at room temperature and rinsed twice in triphosphate buffered saline (TBS) for 5 min. The NovolinkTM Max-Polymer detection system (Novocastra, Newcastle, UK) was used for visualisation, according to the manufacturer’s instructions. After blocking endogenous peroxidase with 3% hydrogen peroxide in methanol for 10 min, sections were incubated overnight at 4°C, with a polyclonal antiserum against *H. pylori* (RBK012; Zytomed, German) which shows immunoreactivity with a wide range of bacteria belonging to the *Helicobacter* genus. Sections were rinsed with TBS between each step of the procedure. Colour was developed for up to 7 min at room temperature with 3,3′-diamino-benzidine (DAB) (Sigma, St. Louis, MO) and sections were then lightly counterstained with haematoxylin, dehydrated and mounted. Positive immunoreactivity was recorded as a distinct golden-brown labelling of the bacteria located on mucosal surface, in gastric pits or glands and in parietal cells.

### Extraction, PCR amplification and sequencing of DNA

DNA was extracted from 5 consecutive slices of 20 μM using a DNeasy Blood and Tissue Kit (Qiagen), according to the manufacturer’s instructions. *Helicobacter* species-specific qPCRs based on a short fragment of the urease A and B genes were developed for the identification of *H. heilmannii s.s.*, *H. felis*, *H. bizzozeronii* and *H. salomonis*. For generation of standards for each qPCR, a large part of the ureAB gene cluster from *H. heilmannii* ASB1 (1224 bp), *H. felis* CS1 (1228 bp), *H. salomonis* R1053 (1224 bp) and *H. bizzozeronii* R1051 (1230 bp) was amplified using primers U430F and U1735R, as described previously [30]. The standard consisted of 10-fold-dilutions starting at 10^8^ PCR amplicons for each 10 μl of reaction mixture. One μl of extracted DNA template was suspended in a 10 μl reaction mixture consisting of 0.25 μl of forward and reverse primers (Table 1), 3.5 μl HPLC water and 5 μl SensiMix™ SYBR No-ROX (Bioline Reagents Ltd, UK). Both standards and samples were run in duplicate on a CFX96™ RT-PCR System with a C1000 Thermal Cycler (Bio-Rad, Hercules CA, USA). The Bio-Rad CFX Manager (version 1.6) software was used for calculation of threshold cycles (Ct)-values and melting curve analysis of amplified DNA. The average values of the duplicates were used for quantification of *Helicobacter* DNA in the tissue samples. To exclude false positive samples, the amplicons from each positive sample were sequenced.

**Table 1:** **List of primers used for qPCR**

**Primer name**	**Nucleotide sequence**	**Specificity**
Hfel_F2	GCT GGT GGC ATC GAT ACG CAT	*H. felis*
Hfel_R2	TTT TTA GAT TAG CGC GTC CGG GA	*H. felis*
HH_FQ	GGC TCT GCG TAG GAC CTG CTA CAG AAG CTC TC	*H. heilmannii s.s.*
HH_RQ	GGC TGT AGG GAT TTG TTG AGG AGA AAT G	*H. heilmannii s.s.*
Hsal_FQ	CTC TTA TGA GTT GGA CTT GGT GCT CAC CAA T	*H. salomonis*
Hsal_RQ	TTT GCC ATC TTT AAT TCC AAT GTC GGC	*H. salomonis*
Hbizz_FQ	AAT CTT TGC GTG GGC CCT GCT ACT GAG	*H. bizzozeronnii*
Hbizz_RQ	CTG GCA AAT GCT GTG GGG ATT TGT TGG	*H. bizzozeronnii*

### Statistical analysis

Pearson’s chi-square test and Fisher’s exact test were used to determine the dependence between two categories. p values < 0.05 were considered as statistically significant. Statistical analysis was performed using the statistical package SPSS 16.0 (SPSS Inc., Produtos e Serviços de Estatística Lda, Lisbon, Portugal).

## Results

A total of 117 gastric samples (66 from the body region and 51 from the antrum region) were analysed.

Among the 69 animals, both gastric regions were available for evaluation in 48 dogs whereas only body or antrum regions were available in 18 and three dogs, respectively.

Of the 69 dogs, only one presented a normal histopathological mucosa with absence of spiral-shaped organisms. A normal gastric mucosa and the presence of spiral-shaped bacteria was observed in two dogs (2.9%). The remaining animals, presented histopathological changes representative of gastritis (66/69 or 95.7%) (Table 2). On the basis of histopathological changes in the gastric mucosa, we diagnosed a mild to moderate chronic gastritis in 88.4% (61/69) affecting the gastric body of 51.5% (34/66) and the antral region of 92.2% (47/51) of the animals.

**Table 2:** **Table summarising the histopathological alterations and colonisation density observed in the canine stomach, according to the positive NHPH species-specific PCR results, positive genus results and negative results**

	**PCR positive results with specific species identification, regardless gastric location (percent&number) (n = 33)**									**Positive results for** ***Helicobacter*** **spp. (percent&number) (n = 27)**	**Negative results (percent & number) (n = 9)**	**p**
	***Hh***	***Hf***	***Hb***	***Hs***	***Hf + Hb***	***Hh + Hs***	***Hh + Hf***	***Hb + Hs***	***Hf + Hb + Hs***			
***Histopathology grading***												
(Day et al., 2008)												
Normal	3.0 (1/33)	0	0	3.0 (1/33)	0	0	0	0	0	7.4 (2/27)	11.1 (1/9)	NS
Mild gastritis	15.2 (5/33)	3.0 (1/33)	3.0 (1/33)	6.1 (2/33)	0	18.2 (6/33)	3.0 (1/33)	3.0 (1/33)	6.1 (2/33)	44.4 (12/27)	33.3 (3/9)	
Moderate gastritis	12.1 (4/33)	3.0 (1/33)	3.0 (1/33)	0	3.0 (1/33)	9.1 (3/33)	0	0	0	48.1 (13/27)	44.4 (4/9)	
Marked gastritis	0	0	0	0	0	6.1 (2/33)	0	0	0	0	11.1 (1/9)	
***Epithelial injury***												
Mild	27.3 (9/33)	3.0 (1/33)	0	9.1 (3/33)	3.0 (1/33)	30.3 (10/33)	0	3.0 1/3(3)	6.1 (2/33)	77.8 (21/27)	33.3 (3/9)	0.003
Moderate	3.0 (1/33)	3.0 (1/33)	6.1 (2/33)	0	0	0	3.0 (1/33)	0	0	14.8 (4/27)	11.1 (1/9)	
***Fibrosis/mucosal atrophy***												
Mild	18.2 (6/33)	6.1 (2/33)	6.1 (2/33)	6.1 (2/33)	0	18.2 (6/33)	0	0	3.0 (1/33)	55.6 (15/27)	33.3 (3/9)	NS
Moderate	3.0 (1/33)	0	0	0	3.0 (1/33)	0	0	0	0	3.7 (1/27)	11.1 (1/9)	
***Intraepithelial lymphocytes***												
Mild	15.2 (5/33)	3.0 (1/33)	6.1 (2/33)	6.1 (2/33)	0	15.2 (5/33)	0	0	6.1 (2/33)	29.6 (8/27)	11.1 (1/9)	0.016
Moderate	3.0 (1/33)	3.0 (1/33)	0	0	3.0 (1/33)	9.1 (3/33)	0	0	0	14.8 (4/27)	0	
***Lymphofollicular hyperplasia***												
Mild	6.1 (2/33)	0	0	0	3.0 (1/33)	0	0	0	0	29.6 (8/27)	11.1 (1/9)	NS
***Bacterial density (based on the IHC results)***											NA	
***+***	0	0	0	0	0	0	0	0	0	14.8 (4/27)		
***++***	3.0 (1/33)	0	0	0	3.0 (1/33)	0	0	0	0	25.9 (7/27)		
***+++***	24.2 (8/33)	6.1 (2/33)	6.1 (2/33)	9.1 (3/33)	0	33.3 (11/33)	3.0 (1/33)	3.0 (1/33)	6.1 (2/33)	51.9 (14/27)		

Legend: *Hh*, *H. heilmannii-like*; *Hf*, *H.felis*; *Hb*, *H. bizzozeronnii*; *Hs*, *H. salomonis.* Bacterial density: +, few organisms; ++, moderate number of organisms; +++, large number of organisms. NA, not applicable. NS, not significant (p > 0.05).

Both mild to moderate epithelial injuries and mild to moderate intraepithelial lymphocyte infiltration were found in 88.4% (61/69).

Gastric mucosal atrophy, glandular nesting or fibrosis was present in 44.9% dogs (31/69). In 18.2% of the animals this alteration was observed in the body region (12/66) and in 47.1% in the antrum region (24/51) of the canine stomach.

Abnormal neutrophilic infiltration was only detected in the stomach antrum of two animals: in one, this alteration was mild and in the other, it was marked and associated with gastric ulceration. Other inflammatory cells, consisting in mild infiltration of mast cells, were observed in the body region of four animals and in the antrum of two animals.

**Figure 1:**
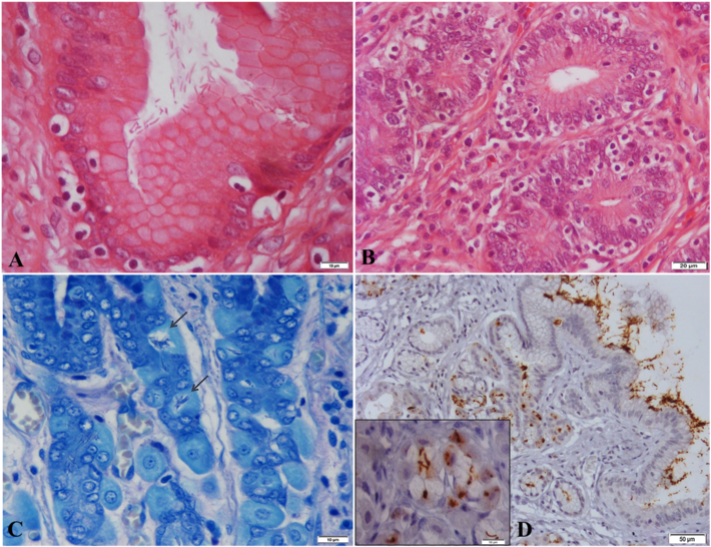
*Helicobacter* spp. in canine stomach. **A)** Numerous spiral-shaped bacteria colonizing the surface epithelium of the gastric pit. HE. Bar = 10 μm; **B)** Note the intraepithelial lymphocyte infiltration within the deeper gastric glands in the antral mucosa of a NHPH-positive dog. HE. Bar = 20 μm. **C)** Presence of NHPH inside the parietal cells of the canine gastric body region (black arrow). MG. Bar = 10 μm; **D)** Large amounts of *Helicobacter* antigen within the superficial gastric mucus and in the lumen of gastric glands in the body region of canine stomach. Immunoperoxidase-diaminobenzidine stain with Mayer’s haematoxylin counterstain. Bar = 50 μm. Inset shows *Helicobacter* antigen within parietal cells, sometimes detectable as well preserved spiral-shaped organisms or brown round dots. Immunoperoxidase-diaminobenzidine stain with Mayer’s haematoxylin counterstain. Bar = 10 μm.

Among all animals, 87.0% were positive (60/69) (Figure 1A) and 13.0% were negative for *Helicobacter* spp. (9/69), irrespective of the test used to detect the bacteria. Regardless of the stomach location, *Helicobacter* spp. were observed using HE, MG and IMC in 65.2% (45/69), 75.4% (52/69) and 82.6% (57/69) of the dogs, respectively. With HE staining, spiral-shaped bacteria were detected in 62.1% of body samples (41/66) and in 70.6% of antral samples (36/51). Utilizing the MG stain, this was 68.2% for the body samples (45/66) and 78.4% for the antral samples (40/51). *Helicobacter* antigen was detected by immunochemistry in 84.9% (56/66) of the body samples and in 80.4% (41/51) of the antral samples (Table 3).

**Table 3:** **Detection of** ***Helicobacter spp.*** **in the different stomach compartments of the canine stomach recurring to different diagnostic methods**

**Gastric region**	**Detection methods**			
	**Positive (percent & number)**			
	**HE**	**MG**	IHC*	**PCR**
Body (n = 66)	62.1 (41/66)	68.2 (45/66)	84.8 (56/66)	37.9 (25/66)
Antrum (n = 51)	70.6 (36/51)	78.4 (40/51)	80.4 (41/51)	51.0 (41/51)

Legend: HE: haematoxylin-eosin; MG: modified Giemsa stain; IHC: immunohistochemistry; PCR: polymerase chain reaction.

*The positive results obtained with IHC did not differ significantly across each stomach region (p > 0.05).

Further identification at species level was performed using *Helicobacter* species-specific qPCRs. *Helicobacter* spp. were detected in 47.8% of the animals (33/69) (Table 3). The majority of the samples were positive in the *H. heilmannii* specific qPCR. However, the amplicons showed only approximately 92% homology with *H. heilmanni s.s.*. Therefore, these cases were reclassified as *H. heilmannii-*like*.*

In 51.5% (17/33) of the positive samples, only one *Helicobacter* species was identified while mixed infections were detected in 48.5% (16/33) (Table 2). *H. heilmannii-*like organisms were the most commonly found (22/33 or 66.7%), being identified in ten dogs as a single infection and in 12 dogs as mixed infections. *H. salomonis* was the second most prevalent species (17/33 or 51.5%) although it was mainly found in association with other NHPH (42%) rather than alone (9.1%). Equal proportions of *H. felis* and *H. bizzozeronnii* were detected (6/33 or 18.2%), either as single (6.3%) or mixed infections (12.1%). Mixed infections with *H. heilmannii*-like and *H. salomonis* were most frequently encountered (33.3%) (Table 2). In the body area, the most frequently identified species was *H. salomonis* (44.0%) whereas in the antrum the most prevalent species was *H. heilmannii-*like (57.7%) (Table 4).

**Table 4:** **Specific** ***Helicobacter*** **species detected by PCR in the different stomach compartments of the canine stomach**

**Specific PCR-** ***Helicobacter spp.*** **positive results**	**Gastric region (percent & number)**	
	**Body**	**Antrum**
	**(n = 25)**	**(n = 26)**
*H. heilmannii-like*	24.0 (6/25)	57.7 (15/26)
*H. salomonis*	44.0 (11/25)	7.7 (2/26)
*H. felis*	8.0 (2/25)	3.8 (1/26)
*H. bizzozeronnii*	4.0 (1/25)	11.5 (3/26)
*H. felis + H. bizzozeronnii*	4.0 (1/25)	3.8 (1/26)
*H. heilmannii-like + H. salomonis*	8.0 (2/25)	11.5 (3/26)
*H. heilmannii-like + H. felis*	4.0 (1/25)	3.8 (1/26)
*H. felis + H. salomonis*	4.0 (1/25)	0

There was a significant correlation between the presence of *Helicobacter* spp. and both mild to moderate epithelial injury and mild to moderate intraepithelial lymphocyte infiltration (Figure 1B) of the canine stomach (p < 0.05). No statistically significantly correlations were found between *Helicobacter* infection and gastric mucosal atrophy or fibrosis, *lamina propria* lymphoplasmacytic infiltration or lymphofollicular hyperplasia. No significant differences were detected regarding the bacterial colonisation density between both stomach regions (p > 0.05).

The number of *Helicobacter*-positive cases detected with the different methods differed significantly (p < 0.05). Positive IHC results did not differ significantly across each stomach region (p > 0.05), while the numbers obtained with HE, GM and qPCR differed significantly between the body and the antrum (p < 0.05).

## Discussion

In this study, a high prevalence of gastritis was observed (95.7%). These results are in agreement with other studies reporting the occurrence of gastritis as a common finding in dogs [14,31,32]. In contrast, gastric erosions or ulcers were rarely found in these animals.

NHPH infection was determined by four methods (HE, MG, IHC and qPCR) and a prevalence of 87.0% in dogs was detected. These results are consistent with those available in the literature, which documented high prevalence of NHPH in the canine gastric mucosa [12,19,33-35]. In our study canine NHPH infection was significantly accompanied by mild to moderate intraepithelial lymphocyte infiltration and mild to moderate gastric epithelial injury, regardless of the stomach location. A clear relationship between canine gastritis and *Helicobacter* infection was, however, not established which is in accordance with results of others [11,14,15,34].

In a previous investigation, the percentage of *Helicobacter*-positive cases detected after HE staining of canine gastric samples was 17.5% [24]. In the current study, all the samples were examined by two pathologists highly experienced in the detection of *Helicobacter* spp*.* organisms after routine staining. This may have played a role in the higher detection rate (65.2%) reported here.

In agreement with previous studies, NHPH were often observed not only in the superficial mucus and within the gastric glands but also intracellularly, in the cytoplasm of parietal cells [31,33,36] (Figure 1C and D). Spiral-shaped organisms present in this particular subcellular location were difficult to detect after HE and MG staining due to the cytoplasm granulation of the parietal cells. In our hands, IHC appeared to be a very valuable technique to identify NHPH within these cells (Figure 1D). Overall, IHC staining showed the highest *Helicobacter*-positive values (82.6%). This finding is in agreement with other results showing that commercially available antibodies against *H. pylori* are useful for the detection of *Helicobacter* spp. in paraffin embedded samples from dog stomachs [24,26,36].

Previous studies reported no statistically significant difference between the detection of *Helicobacter* organisms by IHC and PCR techniques (p > 0.05) [24]. Chung *et al.* (2014) reported that the PCR assay had higher sensitivity and specificity than the other methods [4]. However, in our study, the prevalence rate of *Helicobacter* spp*.* obtained with the qPCR was the lowest (47.8%). Previous investigations have shown that formalin fixation and paraffin embedding hamper PCR analysis [37,38]. Sjödin *et al.* (2011) compared the efficiency of the DNA amplification from fresh (n = 28) and paraffin embedded (n = 28) samples for identification of *Helicobacte*r spp*.* from different organs (feline stomach, duodenum, liver and pancreas) and concluded that the mean value of DNA concentration achieved was higher when obtained from fresh tissues [39]. DNA analysis of paraffin embedded tissue samples using PCR/qPCR may be compromised due to DNA fragmentation, inhibiting substances, or a combination of both. The formalin fixation may indeed cause DNA fragmentation as well as partial destruction of DNA [38,40] and the PCR reactions may also be inhibited by formalin residue [37]. The negative effects of formalin are directly related to the duration of the fixation [40]. Although the samples included in this study were all processed in the same laboratory and therefore subjected to the same standard protocols, duration of fixation may vary between samples due to variation in time interval between sample collection and their arrival at the laboratory.

Different molecular approaches for the identification of NHPH species have been addressed [4,14,24,28]. Techniques based on detection or sequencing of 16S or 23S rRNA-encoding genes can, however, not distinguish between the different canine and feline gastric *Helicobacter* species, whereas tests based on detection or sequencing of the *hsp60* gene, the urease A and B genes or *gyrB* gene allow identification of these bacteria to the species level [9].

In our study, amplicons obtained in the *H. heilmannii* specific qPCR only showed approximately 92% homology with *H. heilmannii,* which so far has only been cultured from the gastric mucosa of cats. Therefore, these cases were reclassified as *H. heilmannii-*like*.* At this moment it is not clear whether *H. heilmannii-*like might represent a new species or a host-adapted variant of *H. heilmannii*.

As previously reported, mixed infections are a common and interesting finding in dogs and cats. Mixed colonisation of the same niche by two or more *Helicobacter* species often occurs and may provide conditions for recombination and genetic exchange between these species. Further studies are necessary to determine if this might play a role in sequence differences in the *ureAB* gene cluster between *H. heilmannii* and *H. heilmannii-*like. An appropriate approach might be *in vitro* isolation and whole genome sequencing of *H. heilmannii*-like from dogs.

According to our results, *H. heilmannii-*like organisms were the most commonly found and *H. salomonis* was mainly found in association with other than NHPH rather than alone. Equal proportions of *H. felis* and *H. bizzozeronnii* were detected, either as single or mixed infections, respectively. Some authors describe *H. felis* as the most commonly found species in dogs [15]. In contrast, in dogs from Finland, Switzerland, the United States and Denmark *H. bizzozeronii* and *H. salomonis* were the most common, followed by *H. felis* and *H. heilmannii* [27,28,33]. In dogs from Belgium, Van den Bulck *et al.* [19] also documented that *H. bizzozeronii* was the most prevalent *Helicobacter* species, as it was identified in 20.0% of the dogs as a single infection and in 50.0% of the dogs as mixed infections. Single infections with *H. felis* or *H. heilmannii*-like were sporadically encountered, while single infections with *H. salomonis* were not identified in any sample [19].

In our study, the prevalence of NHPH was different in both canine stomach regions. Recently, the *in vitro* binding capacity of gastric *Helicobacter* species to canine gastric mucosa was assessed and it was concluded that *H. heilmannii* was the species that adhered the most, followed by *H. felis*, *H. bizzozeronnii* and *H. salomonis*. This tendency was observed in both stomach compartments [41]. Despite its low binding capacity *in vitro*, *H. salomonis* appears to be more effective in colonizing the body stomach of the dog. Regarding the antrum region, the *in vitro* binding assays are in accordance with the results herein obtained and support the high prevalence of *H. heilmannii-like* organisms in this specific location [41].

Given these results and the high proportion of dogs showing the *H. heilmannii-like* and *H. salomonis* bacterial combination, it seems plausible that direct interaction between at least these two species may occur. This high percentage of mixed infections comprising *H. salomonis* may suggest that the colonization capacity of this species in the canine gastric mucosa may be enhanced when associated with other NHPH. However, further studies are needed in order to clarify this hypothesis.

## Conclusions

Taken together, all these results suggest that, despite the high incidence and worldwide distribution of NHPH, the presence of specific Helicobacter species may vary between geographic regions. NHPH infections were significantly accompanied by mild to moderate intraepithelial lymphocyte infiltration and mild to moderate gastric epithelial injury, but a clear relationship between gastritis and Helicobacter infection could not be established.
